# Regeneration of Magnetic Nanoparticles Used in the Removal of Pathogenesis-Related Proteins from White Wines

**DOI:** 10.3390/foods9010001

**Published:** 2019-12-18

**Authors:** Agnieszka Mierczynska-Vasilev, Geridi Qi, Paul Smith, Keren Bindon, Krasimir Vasilev

**Affiliations:** 1 The Australian Wine Research Institute, Waite Precinct, Hartley Grove Cnr Paratoo Road, PO Box 197, Urrbrae (Adelaide) SA 5064, Australia; geridiqi@gmail.com (G.Q.); Paul.Smith@wineaustralia.com (P.S.); keren.bindon@awri.com.au (K.B.); 2 Future Industries Institute, University of South Australia, Mawson Lakes Campus, Mawson Lakes 5095, Australia; krasimir.vasilev@unisa.edu.au; 3 School of Agriculture, Food and Wine, The University of Adelaide, PMB 1, Glen Osmond SA 5064, Australia; 4 Wine Australia, Industry House, National Wine Centre, Cnr Botanic & Hackney Roads, PO Box 2733, Adelaide SA 5000, Australia; 5 School of Engineering, University of South Australia, Mawson Lakes Campus, Mawson Lakes 5095, Australia

**Keywords:** white wine, pathogenesis-related proteins, magnetic nanoparticles, protein removal, regeneration

## Abstract

Protein haze remains a serious problem for the wine industry and requires costly bentonite treatment, leading to significant wine volume loss. Recently developed magnetic separation technology that allows a fast and efficient separation of haze proteins from wine shows promise for the development of an alternative method for white wine fining. The key purpose of this study was to understand the potential of the nanoparticles to be reused in multiple fining and regeneration cycles. Bare and acrylic-acid-based plasma polymer coated magnetic nanoparticles were cleaned with water, 10% SDS/water and acetone/water solution after each adsorption cycle to investigate their restored efficiency in removing pathogenesis-related proteins from three unfined white wines. The concentrations of metals, acids and phenolics were monitored to determine changes in the concentration of these essential wine constituents. The regeneration study verified that the acrylic acid plasma-coated magnetic nanoparticles, which underwent ten successive adsorption-desorption processes, still retained close to the original removal capacity for haze proteins from wines when 10% SDS solution and water were used for surface regeneration. In addition, the concentrations of organic acids and wine phenolic content remained almost unchanged, which are important indicators for the retention of the original wine composition.

## 1. Introduction

Protein instability, which leads to turbidity in white wines, is a serious quality consideration, since consumers perceive haze in wines as a defect [1,2]. Protein haze is caused by pathogenesis-related proteins [3] becoming unstable after bottling and later especially when exposed to elevated temperatures during storage and transportation [4]. Wine temperature can occasionally reach 50–60 °C in shipping containers (if the outside temperature is 40 °C, the inside of an uninsulated container can be as high as 60 °C [5]) or in vehicles on a warm day [6]. This might result in the accelerated ageing of the wine and an acceleration of the hazing phenomenon. The content of haze-forming proteins in white wines can vary from 10 to 400 mg/L [7]. The most abundant classes of haze-forming proteins in white wine are chitinases and thaumatin-like proteins (TLPs) [3]. These proteins are small and compact (the molecular weight of TLP is 15–25 kDa, chitinase is 25–30 kDa), have a globular structure [8] and are positively charged at wine pH. Conventionally, winemakers use bentonite fining to remove proteins and hence prevent haze formation. It is a treatment that works well but has several disadvantages. Bentonite is a clay and is added as a suspension in water. It is not trivial to prepare the slurry or to add it to wine, and it needs to be prepared reasonably concentrated as there are limits to the amount of water that can be added to wine. Whilst bentonite is relatively specific for wines, proteins, colour, aroma and texture compounds can potentially also be removed [4,9]. Furthermore, being a clay, bentonite swells in the wine solution and does not settle compactly, which leads to an occlusion of wine in the lees. Depending upon subsequent processing steps, 3%–20% of the wine volume is lost together with the bentonite lees [9,10]. Importantly, bentonite cannot be recycled, and hence, there are labour and waste disposal issues associated with its use. The overall estimate of loss caused by bentonite fining in Australia is around $100 M annually. Globally, these losses equate to approximately $1 billion per year [11]. Alternative solutions to bentonite fining such as various other adsorbents [12,13], proteases [14,15,16], flash pasteurisation [17], magnetic nanoparticles [18,19] and more recently, natural zeolites [20] have been proposed.

Recently [18], we reported a new magnetic separation technology that allows for selective removal of pathogenesis-related proteins from wines. The technology is based on plasma coating of magnetic nanoparticles with polymers, thus creating carefully defined surface functionality. After these plasma-coated magnetic nanoparticles have been placed in heat-unstable wines, the proteins bind to the surface coating and can then be removed from the wine using a magnet. The technology is simple and has been shown to be very effective in removing proteins from a range of wine types [18].

The aim of the current study was to demonstrate the capacity to regenerate the plasma polymer coated magnetic nanoparticles for re-application, which is important from a technology adoption perspective, especially given that nanoparticles are more expensive then bentonite. We hypothesised that protein adsorption onto the nanoparticle surface would be a reversible process, and thus, regeneration and reuse of magnetic nanoparticle could be possible. The effect of three different protein cleaning solvents (water, 10% SDS/water and acetone/water) on the removal of pathogenesis-related proteins from the surface of magnetic nanoparticles was evaluated. Effects on protein concentration, phenolic content, organic acid concentration and elemental composition in the treated wines were also characterised in detail.

## 2. Materials and Methods

### 2.1. Wines

Unfined 2017 Sauvignon blanc (SAB), Semillon (SEM) and Chardonnay (CHA) wines used in the study were supplied by Accolade Wines, Reynella, South Australia. Table S1 (Supplementary Material) presents the basic chemical data for the three wines.

### 2.2. Magnetic Nanoparticles

Magnetic nanoparticles (MNPs) (316 stainless steel grade) were supplied by SkySpring Nanomaterials, Inc., Huston, TX, USA. Bare and acrylic acid plasma coated magnetic nanoparticles were used. The bulk and surface elemental composition as well as morphology of magnetic nanoparticles used was specified in a previously published work [18,19]. Plasma polymerisation of acrylic acid was carried out in a bell-chamber reactor as described previously [18,19].

### 2.3. Adsorption and Desorption of Haze-Forming Proteins in Wine by Magnetic Nanoparticles

The adsorption of pathogenesis-related proteins by bare and acrylic acid plasma functionalised MNPs was investigated in triplicate in three unfined wines. A 1.7 vol.% dose was used, which corresponded to 13 g/L of the bare (batch 1) and acrylic-acid-functionalised MNPs (batch 2) in 50 mL centrifuge tubes containing wine and mixed on a suspension mixer for 30 min at room temperature. Then, on batch bases, the MNPs with adsorbed proteins were separated from the wine with a hand-held permanent magnet and samples for protein, organic acids, phenolics and metal analysis were withdrawn from the treated wines. To investigate the removal of adsorbed proteins from the surface of bare and coated magnetic nanoparticles, three solvent types were compared: pure Milli-Q water and Milli-Q water containing 10% sodium dodecyl sulphate solution (SDS) (Sigma-Aldrich, Darmstadt, Germany) or acetone (Sigma-Aldrich, purity ≥99.5%). The desorption study was performed by mixing 13.3 g of wine-treated MNPs with cleaning solution (Milli-Q water, 10% SDS or acetone) for 30 min on a suspension mixer. Cleaning with Milli-Q water was repeated 6 times (6 × 30 min with Milli-Q water), while cleaning with 10% SDS solution and acetone was repeated three times. After SDS and acetone treatment, the MNP samples were rinsed with water three times to ensure none of the two solvents was left on the MNPs surface. After each cleaning cycle, suspensions were placed in the oven for drying to a constant weight (2 h) at 60 °C and cooled to room temperature for reuse. The consecutive adsorption-desorption process was carried out ten times. As a preliminary study, the removal of haze-forming proteins from MNPs and the corresponding pH change in the wash solution was determined for successive wash steps for the water and 10% SDS treatments. For the acetone wash treatment, the water from the final wash steps was analysed for haze-forming protein.

### 2.4. Wine Analysis

HPLC (Agilent Technology 1200 system, Santa Clara, CA, USA) was used to analyse proteins and organic acids in wine before and after treatment. The concentration of haze-forming wine proteins was measured according to a previously published method [18]. The concentration of organic acids (citric, tartaric, malic, succinic and lactic) was measured as described previously by Marangon et al. [21]. Phenolic content in wines before and after treatment with bare and plasma-coated MNPs was measured on Cary 60 UV-vis spectrometer (Agilent Technologies, Santa Clara, CA, USA). Absorbance in the spectral range of 280 to 420 nm was measured. Metal content in wine was determined by inductively coupled plasma–optical emission spectrometry (ICP-OES) performed by AWRI Commercial Services. All study samples were analysed without dilution.

## 3. Results and Discussions

### 3.1. Protein Content in Wines before and after Treatment with Magnetic Nanoparticles

**Figure 1:**
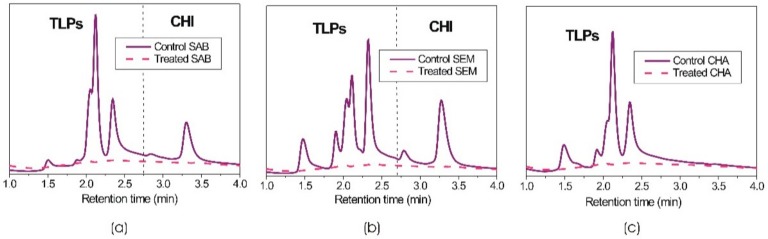
HPLC chromatograms of wines before and after treatment with acrylic acid plasma coated magnetic nanoparticles (**a**) Sauvignon blanc, (**b**) Semillon and (**c**) Chardonnay wine; (thaumatin-like proteins (TLPs), chitinases (CHI)).

In the first component of the study, our aim was to investigate the adsorption capability of MNPs for the removal of pathogenesis-related proteins from various unfined wines in ten consecutive adsorption-desorption cycles. The initial protein concentration in the three investigated wines was determined by high-performance liquid chromatography (HPLC). Figure 1 shows the HPLC chromatograms for the control wine samples and the same wines after treatment with 1.66 vol.% of acrylic acid plasma coated MNPs. The initial protein concentration in Sauvignon blanc, Semillon and Chardonnay wines was 130, 205 and 116 mg/L, respectively. Whereas in Sauvignon blanc and Semillon wines, both chitinases and thaumatin-like proteins (TLPs) were present, there were no chitinases in the Chardonnay wine. After treatment with acrylic acid plasma coated MNPs, all pathogenesis-related proteins had been removed, which was consistent with our previously published reports [18,19].

### 3.2. Effect of Magnetic Nanoparticles on Metal Content

The impact of MNP treatment on the metal content of wine was also investigated. Table 1 shows the metal content in control Sauvignon blanc and after treatment with bare and acrylic acid plasma coated MNPs.

A striking result was the significant decrease of potassium in SAB wine after treatment with MNPs. The starting concentration of potassium for SAB was 829 mg/L. After treatment with bare and acrylic-acid-coated MNPs, the value decreased to 86 and 84 mg/L, respectively. This significant reduction in the potassium content has an additional benefit to winemaking as it might aid cold stabilisation, a necessary production step to prevent the crystallisation of potassium tartrate salts in a finished wine during storage. Currently, this is achieved through an extensive cooling process where wine is held below 4 °C, followed by filtration. As reported in the literature by Wyss and Cuénat [22], improved tartrate stability can be achieved by decreasing the content of potassium. In the current study, the potassium concentration in wine was reduced by more than 80% following treatment with MNPs. Thus, the magnetic separation technology reported here could potentially accomplish removal of haze protein and reduction of potassium content in a single step. In general, SAB wine treated with bare and AcrA/MNPs had less K, Ca, Mg, Na, Zn and Sr. The only element which increased in content after MNP treatment was Mn. The amount of Fe and Cu did not change significantly. However, it is worth mentioning that the concentrations of all these metals were within the range common for wines [23].

### 3.3. Desorption of Wine Proteins from Magnetic Nanoparticle Surface as a Function of the Cleaning Solvent and Wash Cycle

To investigate the desorption of wine proteins from the surface of MNPs and to determine the number of wash cycles required for complete protein removal and regeneration of MNPs, studies were performed to quantify the amount of protein present in the cleaning solvent after each wash cycle.

**Figure 2:**
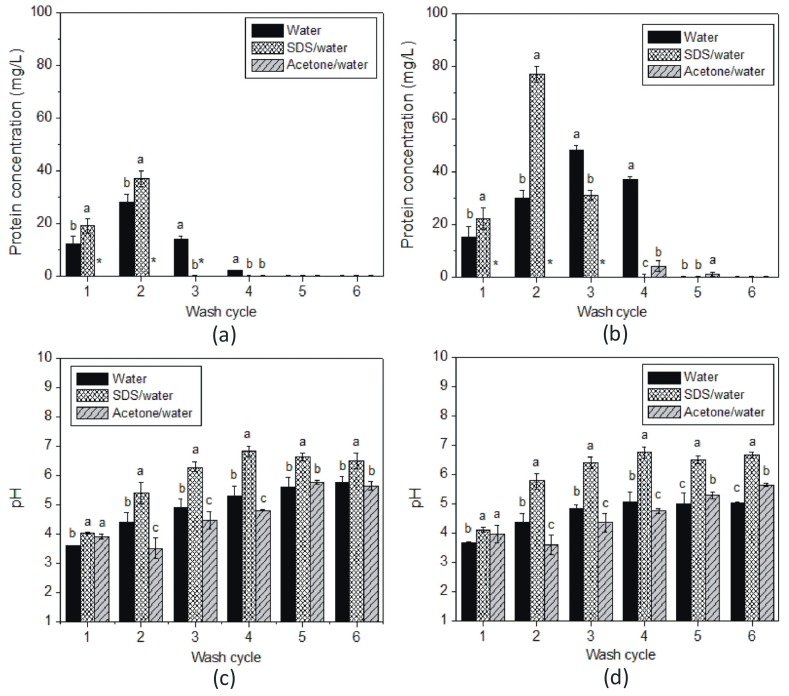
Protein concentration in the supernatant as a function of wash cycle for: (**a**) bare MNPs and (**b**) plasma-coated MNPs; pH of the supernatant as a function of wash cycle for: (**c**) bare MNPs and (**d**) plasma-coated MNPs. * The amount of protein was not measured in pure acetone due to insolubility and/or incomplete recovery from the solvent. Significant differences between solvents on protein removal or pH was assessed by Student’s *t* test at each wash step. Mean values with a different letter indicate significant differences (*p* < 0.05).

Three different solvents were used to separate the proteins from MNP surface: Milli-Q water, 10% sodium dodecyl sulphate (SDS) solution and acetone. Water was used because of its convenience and low cost. SDS was chosen as one of the best solvents for removing biological matter. including proteins adsorbed to biomaterial surfaces [24]. Acetone was selected because of its capacity to clean a wide range of surface contaminations. In this experiment, the SAB wine with initial protein concentration of 130 mg/L was used. Figure 2a,b shows the amount of protein measured by HPLC in the supernatant as a function of wash cycle for bare and plasma-coated MNPs, respectively.

**Figure 3:**
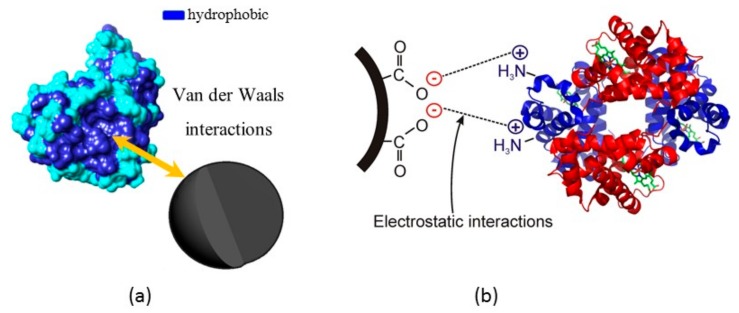
Schematic of a possible mechanism for protein adsorption (**a**) on the bare magnetic nanoparticle surface and (**b**) on acrylic acid plasma coated magnetic nanoparticle surface.

As stated previously, cleaning with Milli-Q water was repeated six times, whereas cleaning with 10% SDS solution and acetone was repeated three times. In addition, after SDS and acetone treatment, the MNP samples were washed with Milli-Q-water three times to ensure none of the two solvents remained on the surface. Since proteins are likely to adsorb via van der Waals forces to bare MNPs (as depicted in Figure 3a), it would not be expected that proteins could be effectively separated from the bare MNP surface via any of the three solvents investigated [18], and for that reason, only ~56 mg/L out of 130 mg/L of wine protein was recovered, as presented in Figure 2a. As reported previously [18], proteins are attracted to acrylic acid plasma coated MNPs via longer range electrostatic interactions, as depicted in Figure 3b, and therefore, they are more efficient in the adsorption of proteins from wines. Within the pH range of the white wines investigated in this study (3.4, 3.4 and 3.3 for SAB, SEM and CHA, respectively), the coated nanoparticles would have maintained a small negative charge on the surface [25] which would have been sufficient to facilitate binding to positively-charged wine proteins. The situation potentially changed during washing with water, SDS and acetone. These three solvents have a higher pH (pH of water and acetone is 7, while pH of 10% SDS is 8.3), which resulted in a switching the protein charge from positive to negative (pI ~4.1 and 4.6 [8,26]). In this new environment, the proteins and negatively charged acrylic acid plasma coating (−35 mV at pH 7 [25]) would be expected to repulse one other, leading to desorption from the surface of the nanoparticles.

After the first wash cycle in water and SDS, the amount of protein transferred from the nanoparticle surface to the supernatant was approximately equal, 15 and 20 mg/L for bare and acrylic acid plasma coated MNPs, respectively. As can be seen from Figure 2, the second wash was more effective for SDS as the amount of protein released from MNPs and measured in the supernatant was 27 and 77 mg/L for bare and acrylic acid plasma coated MNPs, respectively. The increased protein concentration was likely to be due to the increased pH of the supernatant. Figure 2c,d shows the pH of the supernatant as a function of wash cycle. As can be seen from the Figure 2, for the second SDS wash, the pH increased above the pI of the wine protein, at 5.3 and 5.8 for bare and plasma-coated MNPs, respectively. Water was more effective in the third wash for plasma-coated MNPs when the pH increased to 4.8. In consecutive washes, the pH increased further, and the desorption of wine proteins became more effective. From this preliminary assessment, it was concluded that six washes were sufficient to remove all the protein from the surface of the MNPs. Four washing cycles in the case of water and five for acetone were required for total protein recovery, respectively, while for SDS, three washes were sufficient. Depending on the solvent used, for 1 L of wine, 4 to 6 L of solvent was required during the nanoparticles regeneration procedure.

### 3.4. Effect of the Cleaning Solvent Used for Regeneration on Performance of MNPs in Wine Protein Removal

**Figure 4:**
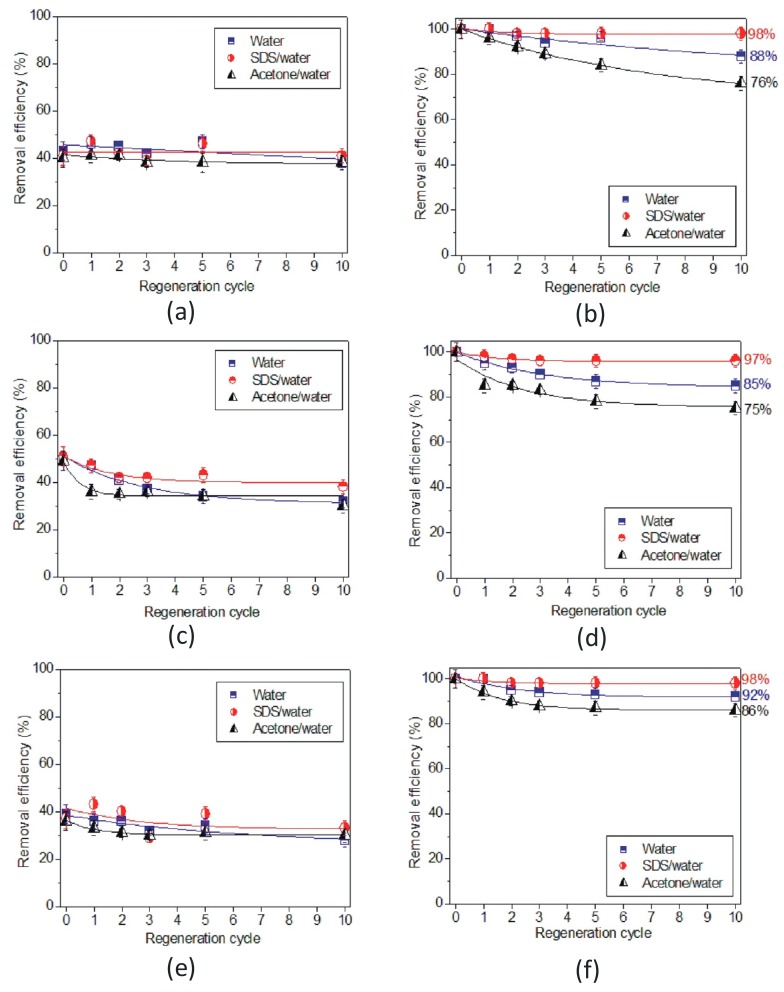
Protein removal efficiency of Milli-Q water, 10% SDS/water and acetone/water for: (**a**) Sauvignon blanc, (**c**) Semillon and Chardonnay wine after treatment with bare, agnetic nanoparticles (MNPs); (**b**) Sauvignon blanc, (**d**) Semillon and (**f**) Chardonnay wine treated with acrylic acid plasma coated magnetic nanoparticles (AcrA-MNPs).

For MNP technology to be practically viable, it was necessary to demonstrate that the nanoparticles could be regenerated for multiple re-applications. Figure 4a–c shows the percentage of removed proteins from bare MNP surfaces as a function of regeneration cycle for three different cleaning solvents, and for three different unfined wines. As can be seen from Figure 4a–c, in the case of bare MNPs, protein removal was low, at only about 30%–40%. Overall, there was no significant difference in protein removal between cleaning solvents for SAB and CHA wines. For SEM wine, 10% SDS cleaning solution appeared to be more efficient (although only marginally) to remove MNP-bound haze proteins compared with those regenerated with water and acetone.

In contrast to what was observed for bare MNPs, acrylic acid plasma polymer coated MNPs more efficiently removed wine protein, as was discussed previously (Figure 4b,d,f). The percentage of protein removal in relation to the regeneration cycles for coated MNPs decreased gradually for all three cleaning solvents (R2 for fittings are provided in Table S2 in Supplementary Material). The 10% SDS solution was the most effective solvent in the regeneration of MNPs and retained the protein removal efficiency after the tenth regeneration cycle for all three wines. Removal percentages were 98%, 97% and 98% of protein removal for the Sauvignon blanc, Semillon and Chardonnay wines at the final regeneration step, respectively. Additionally, cleaning the surface of coated MNPs with Milli-Q water alone resulted in the retention of a high protein removal efficiency: at 88%, 85% and 92% after the tenth regeneration cycle for SAB, SEM and CHA wine, respectively. The least efficient solvent was acetone with 76%, 75% and 86% protein removal from SAB, SEM and CHA wine, respectively, at the tenth regeneration cycle (for statistical analysis, please refer to Supplementary Material Tables S3–S8).

### 3.5. Phenolic Composition of the Wines

**Figure 5:**
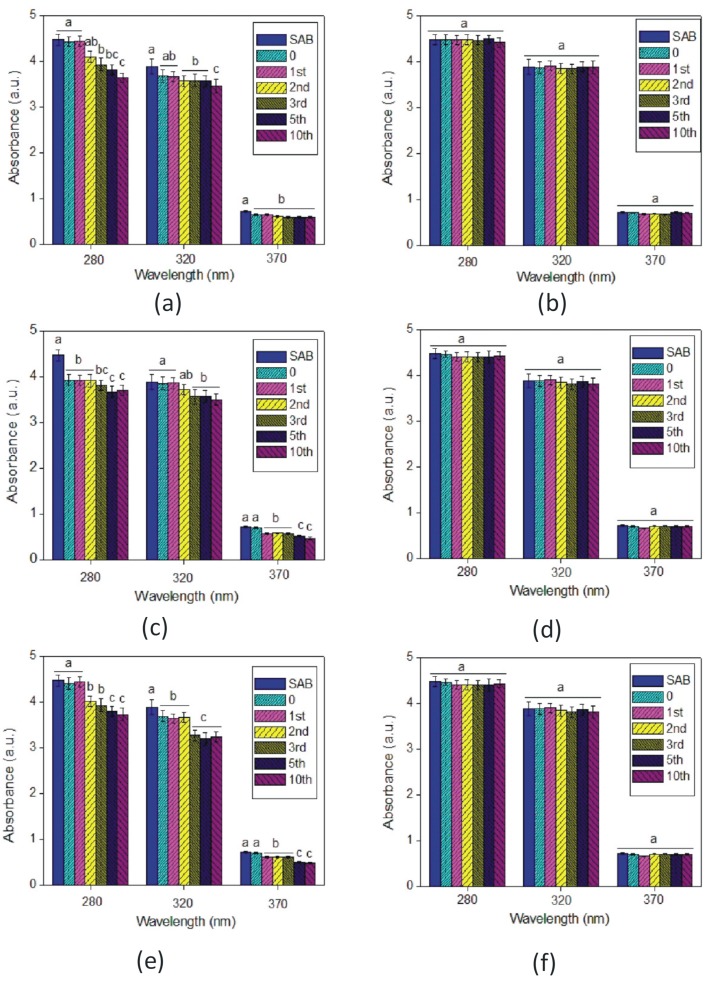
Phenolic content of Sauvignon blanc wine before and after treatment with bare (**a**,**c**,**e**) and acrylic acid plasma coated magnetic nanoparticles (**b**,**d**,**f**). Three cleaning solvents were used: (**a**,**b**) Milli-Q water, (**c**,**d**) SDS and (**e**,**f**) acetone. Data significance was assessed by Student’s *t* test. Mean values with a different letter were significantly different (*p* < 0.05).

Absorption in the spectral range of 280 to 420 nm was measured to compare the phenolic content in wines before and after treatment with MNPs. Figure 5a,c,e shows the spectral fingerprints of SAB wine before and after treatment with bare MNPs. The 280 and 320 nm wavelengths give an indication of the concentration of total phenolics [27], and low molecular weight phenolics such as hydroxycinnamates [28] and flavanols are detected at 370 nm. As presented in Figure 5a,c,e, there were differences in phenolic composition for control wines in comparison with wine after treatment with MNPs regardless of the cleaning solvent used. A decrease in absorbance values was observed when wine was treated with bare MNPs. The results suggest that phenolics can potentially bind to bare MNPs. Phenolic content was also measured for SEM and CHA wines treated with bare MNPs. As shown in the Supplementary Material (Figures S1 and S2), similar trends were observed for SEM and CHA wines treated with bare MNPs as found for the SAB wines.

Contrary to the results found for bare MNPs, the absorbance spectra of SAB wine before and after treatment with acrylic acid plasma coated MNPs were very similar, as shown in Figure 5. This indicated that, whether fresh or regenerated, the plasma-treated MNPs did not alter the phenolic (UV) signal of the SAB wine, in comparison with bare MNPs. Phenolic content was also measured for the other two wines, i.e., SEM and CHA, yielding similar results (Supplementary Material Figures S1 and S2).

### 3.6. Concentration of Organic Acids

**Figure 6:**
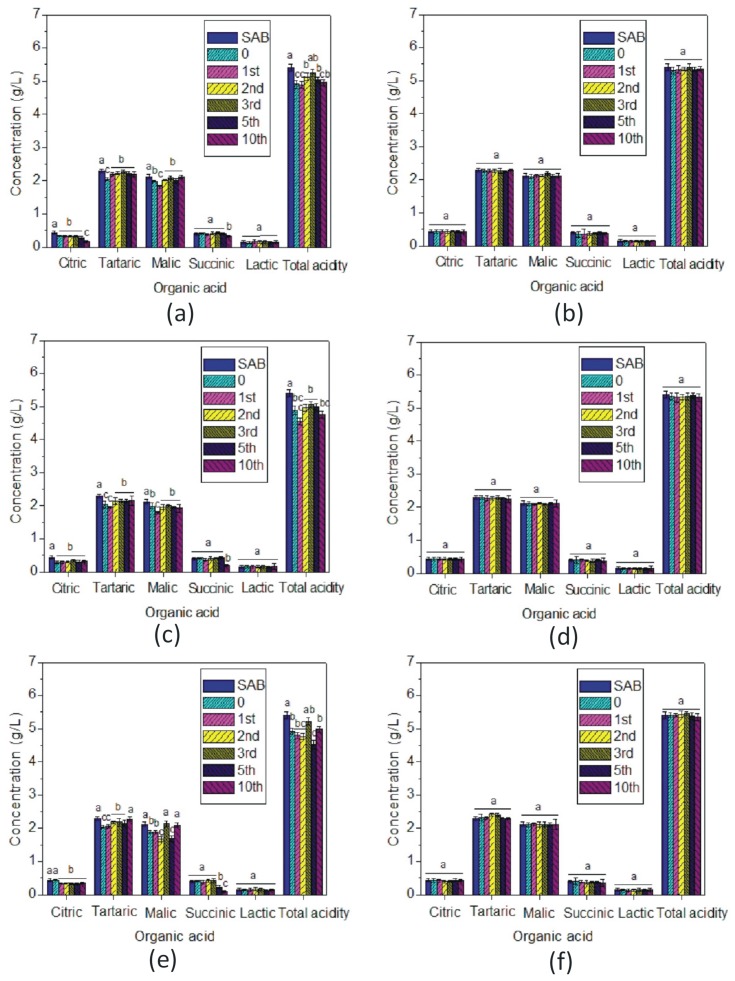
Organic acids concentration of Sauvignon blanc wine before and after treatment with bare and acrylic acid plasma coated magnetic nanoparticles. Three cleaning solutions were used: (**a**,**b**) Milli-Q water, (**c**,**d**) SDS and (**e**,**f**) acetone. Data significance was assessed by Student’s *t* test. Mean values with a different letter were significantly different (*p* < 0.05).

Organic acid profiling was used to study the effect of the MNP treatments on major wine acids. The concentration of organic acids (citric, tartaric, malic, succinic and lactic) was analysed. Figure 6a,c,e presents the concentration of organic acids in SAB wine before and after treatment with bare MNPs. There was a reduction in citric, tartaric, malic and succinic acid concentration for treated SAB wine when MNP regeneration with water and SDS was performed (Figure 6a,c), but the response changed slightly when acetone was used for MNP cleaning. On the other hand, SAB treated with acrylic acid plasma coated MNPs exhibited no significant difference in the amount of organic acids before and after treatment regardless of the cleaning solvent used (Figure 6b,d,f). As was observed for wine phenolics, this indicates a promising outcome for the use of coated MNPs in protein fining, since other important nonvolatile wine components are unlikely to be affected by the treatment or regeneration approach. Furthermore, the efficiency of coated MNPs was consistent across the two remaining two wines (SEM and CHA) used in the study, indicating that the effects were not dependent upon the wine matrix (Figures S3 and S4). Conversely, the selectivity of bare MNPs for the different organic acids varied when bare MNPs were used in the SEM and CHA wines (Figures S3 and S4), compared with the SAB wine discussed previously. The response was also found to be inconsistent across the different regeneration treatments. Generally, bare MNPs consistently decreased total acidity in the wine as the cleaning cycles progressed, independently of the regeneration solvent used. Therefore, in comparison with the coated MNPs, it could be concluded that the use of bare MNPs as protein fining agents could potentially introduce greater, less predictable changes to the wine matrix.

## 4. Conclusions

In this work, the potential for the development of an MNP-based separation technology for the removal of haze proteins from white wines was further elucidated. Considering the practical implementation of the MNP fining process, it was important to understand the potential of the nanoparticles to be regenerated and reused. Regeneration studies were carried out using bare and acrylic acid plasma coated MNPs in ten consecutive adsorption-desorption processes utilising three different solvents (Milli-Q water, 10% SDS solution and acetone). Analysis of other important wine constituents such as metals, organic acids and phenolics was also conducted in order to determine the impact of the MNP technology on the key attributes relevant to the maintenance of the quality of the wines. Bare MNPs were not effective in removing pathogenesis-related proteins from wines and could not be successfully regenerated via the three solvents selected for this study. The latter could be attributed to the nature of the interactions between the proteins and the bare nanoparticle surface. In addition, bare MNPs lead to a variable response in terms of the amount of key wine constituents such as organic acids and phenolics, which are important to control in winemaking. In contrast, acrylic acid plasma coated MNPs exhibited a high protein removal capacity and good reusability within ten successive adsorption-desorption processes. In all cases, 10% SDS/water and water performed very well, showing a very high protein removal efficiency (~98% for 10% SDS/water and ~85%–92% for water) after ten successive adsorption-desorption processes. Importantly, the concentrations of organic acids and wine phenolic content remained almost unchanged, suggesting that key wine constituents would be preserved. The adsorption-desorption results suggested that the acrylic acid plasma coated MNP surface could be regenerated successfully with both 10% SDS and water and therefore have potential for commercial application.

## Figures and Tables

**Table 1:** Metal concentration of Sauvignon blanc (SAB) before and after treatment with bare and acrylic acid plasma coated magnetic nanoparticles.

Element (mg/L) *	Control SAB (pH 3.39)	SAB + Bare MNPs	SAB + AcrA/MNPs
K	829 ± 50 a	86 ± 15 b	84 ± 16 b
Ca	53 ± 5 a	<30 b	<30 b
Mg	94 ± 5 a	<50 b	<50 b
Na	19 ± 1 a	<10 b	<10 b
Mn	1.4 ± 0.2 c	3.2 ± 0.5 b	4.4 ± 0.8 a
Cu	<0.1 a	0.1 a	0.1 a
Zn	0.89 ± 0.1 a	0.32 ± 0.2 b	0.31 ± 0.1 b
Fe	0.6 ± 0.2 b	0.9 ± 0.5 a	0.8 ± 0.2 a
Sr	1.43 ± 0.3 a	0.16 ± 0.1 b	0.15 ± 0.1 b

Data represent the average of three independently prepared and tested samples. * Cd, Cr, Co, Pb, Ni and Se were measured, but their concentrations were below detection limit. Data significance was assessed by Student’s *t* test. Mean values with a different letter were significantly different (*p* < 0.05).

## Supplementary Materials

The following are available online at https://www.mdpi.com/2304-8158/9/1/1/s1, Table S1: Basic chemical analysis of the wines used in this study. Table S2: R2 for all the samples presented on Figure 4. Table S3: Protein removal efficiency of Milli-Q water, 10% SDS/water and acetone/water for Sauvignon blanc wine treated with bare magnetic nanoparticles. Table S4: Protein removal efficiency of Milli-Q water, 10% SDS/water and acetone/water for Semillon wine treated with bare magnetic nanoparticles. Table S5: Protein removal efficiency of Milli-Q water, 10% SDS/water and acetone/water for Chardonnay wine treated with bare magnetic nanoparticles. Table S6: Protein removal efficiency of Milli-Q water, 10% SDS/water and acetone/water for Sauvignon blanc wine treated with acrylic acid plasma coated magnetic nanoparticles. Table S7: Protein removal efficiency of Milli-Q water, 10% SDS/water and acetone/water for Semillon wine treated with acrylic acid plasma coated magnetic nanoparticles. Table S8: Protein removal efficiency of Milli-Q water, 10% SDS/water and acetone/water for Chardonnay wine treated with acrylic acid plasma coated magnetic nanoparticles. Figure S1: Phenolic content of Semillon wine before and after treatment with bare and acrylic acid plasma coated magnetic nanoparticles. Figure S2: Phenolic content of Chardonnay wine before and after treatment with bare and acrylic acid plasma coated magnetic nanoparticles. Figure S3: Organic acid concentration of Semillon wine before and after treatment with bare and acrylic acid plasma coated magnetic nanoparticles. Figure S4: Organic acids concentration of Chardonnay wine before and after treatment with bare and acrylic acid plasma coated magnetic nanoparticles.
