# Supplementary material for: Regeneration of Magnetic Nanoparticles Used in the Removal of Pathogenesis-Related Proteins from White Wines
Source: Foods. 2019 Dec 18;9(1):1. doi: 10.3390/foods9010001 (PMC7022247; doi:10.3390/foods9010001)
Supplement: Supplementary file 1 [file foods-09-00001-s001.pdf]

Supplementary Materials

Regeneration of magnetic nanoparticles used in the removal of pathogenesis-related proteins from white wines

Agnieszka Mierczynska-Vasilev[1,2], Geridi Qi[1,3], Paul Smith [1,4], Keren Bindon[1], Krasimir Vasilev[2,5]*

The Australian Wine Research Institute, Waite Precinct, Hartley Grove Cnr Paratoo Road, Urrbrae (Adelaide) SA 5064, PO Box 197, Australia;

[2] Future Industries Institute, University of South Australia, Mawson Lakes Campus, Mawson Lakes 5095, Australia;

[3] School of Agriculture, Food and Wine, The University of Adelaide, PMB 1, Glen Osmond, SA 5064, Australia

[4] Wine Australia, Industry House, National Wine Centre, Cnr Botanic & Hackney Roads, Adelaide SA 5000, PO Box 2733, Australia

[5] School of Engineering, University of South Australia, Mawson Lakes Campus, Mawson Lakes 5095, Australia

Table S1. Basic chemical analysis of the wines used in this study.

Analysis	SAB	SEM	CHA
Alcohol (% v/v)	13.1	12.8	12.6
Glucose + Fructose (g/L)	0.8	1.1	0.6
Malic acid (g/L)	1.61	1.29	1.46
pH	3.39	3.35	3.33
Specific gravity	0.990	0.991	0.991
Sulfur Dioxide (free) (mg/L)	23	35	14
Sulfur Dioxide (total) (mg/L)	135	132	70
Titratable acid pH 7.0 (g/L)	5.4	5.5	7.1
Titratable acid pH 8.2 (g/L)	5.9	5.9	7.4
Volatile acidity as acetic acid (g/L)	0.29	<0.25	0.3
Protein content (mg/L)	130	205	116

SAB = Sauvignon Blanc, SEM = Semillon and CHA = Chardonnay

Table S2. R2 for all the samples presented on Figure 4.

Wine	Cleaning solvent	R2*
CHA	Water	0.88
	SDS/Water	0.72
	Acetone/Water	0.99
SEM	Water	0.99
	SDS/Water	0.82
	Acetone/Water	0.88
SAB	Water	0.83
	SDS/Water	0.72
	Acetone/Water	0.99

*data were fitted with the first order exponential decay.

Table S3. Protein removal efficiency of Milli-Q water, 10% SDS/water and acetone/water for Sauvignon blanc wine treated with bare magnetic nanoparticles.

Sample	Cleaning cycle for water	Removal efficiency (%)	Variation	Significance
**Bare MNPs+ SAB**	**0**	**43 ± 4**		
	1st	46 ± 3	+ 3	ns
	2nd	45 ± 2	+ 2	ns
	3rd	42 ± 2	- 1	ns
	5th	47 ± 3	+4	***
	10th	38 ± 3	- 5	***
	**Cleaning cycle for SDS/water**			
Bare MNPs+ SAB	0	41 ± 4		
	1st	47 ± 3	+ 6	***
	2nd	42 ± 2	+ 1	ns
	3rd	39 ± 2	- 2	ns
	5th	46 ± 3	+ 5	***
	10th	41 ± 3	0	ns
	**Cleaning cycle**			

	for acetone/water			
Bare MNPs+ SAB	0	40 ± 4		
	1st	41 ± 3	+ 1	ns
	2nd	41 ± 2	+ 1	ns
	3rd	38 ± 2	- 2	ns
	5th	38 ± 4	- 2	ns
	10th	38 ± 3	- 2	ns

All data are expressed as mean ± standard deviation (n=3).

According to Student t-test *,**,*** and ns indicate significance at P < 0.001, 0.01, 0.05 and not significant, respectively.

Table S4. Protein removal efficiency of Milli-Q water, 10% SDS/water and acetone/water for Semillon wine treated with bare magnetic nanoparticles.

**Sample**	**Cleaning cycle for water**	**Removal efficiency (%)**	**Variation**	**Significance**
Bare MNPs+ SEM	0	49 ± 4		
	1st	47 ± 3	- 2	ns
	2nd	41 ± 2	- 8	***
	3rd	37 ± 2	- 12	***
	5th	34 ± 3	- 15	***
	10th	32 ± 3	- 17	***
	**Cleaning cycle for SDS/water**			
Bare MNPs+ SEM	0	51 ± 4		
	1st	47 ± 3	- 4	**
	2nd	42 ± 2	- 9	***
	3rd	42 ± 2	- 9	***
	5th	43 ± 3	- 8	***
	10th	38 ± 3	- 13	***
	**Cleaning cycle for acetone/water**			
Bare MNPs+ SEM	0	49 ± 4		
	1st	36 ± 3	- 13	***
	2nd	35 ± 2	- 14	***
	3rd	36 ± 2	- 13	***
	5th	34 ± 3	- 15	***
	10th	30 ± 3	- 19	***

All data are expressed as mean ± standard deviation (n=3).

According to Student t-test *,**,*** and ns indicate significance at P < 0.001, 0.01, 0.05 and not significant, respectively.

Table S5. Protein removal efficiency of Milli-Q water, 10% SDS/water and acetone/water for Chardonnay wine treated with bare magnetic nanoparticles.

Sample	Cleaning cycle for water	Removal efficiency (%)	Variation	Significance
Bare MNPs+ CHA	0	39 ± 4		
	1st	36 ± 3	- 3	ns
	2nd	36 ± 2	- 3	ns
	3rd	32 ± 2	- 7	**
	5th	34 ± 3	- 5	**
	10th	28 ± 3	- 11	***
	**Cleaning cycle for SDS/water**			
Bare MNPs+ CHA	0	37 ± 4		
	1st	43 ± 3	+ 6	***
	2nd	40 ± 2	+ 3	**
	3rd	29 ± 2	- 8	***
	5th	39 ± 3	+ 2	ns
	10th	33 ± 3	- 4	**
	**Cleaning cycle for acetone/water**			
Bare MNPs+ CHA	0	36 ± 4		
	1st	33 ± 3	- 3	**
	2nd	31 ± 2	- 5	***
	3rd	30 ± 2	- 6	***
	5th	31 ± 3	- 5	***
	10th	30 ± 3	- 6	***

All data are expressed as mean ± standard deviation (n=3).

According to Student t-test *,**,*** and ns indicate significance at P < 0.001, 0.01, 0.05 and not significant, respectively.

Table S6. Protein removal efficiency of Milli-Q water, 10% SDS/water and acetone/water for Sauvignon blanc wine treated with acrylic-acid plasma coated magnetic nanoparticles.

Sample	Cleaning cycle for water	Removal efficiency (%)	Variation	Significance
AcrA-MNPs+ SAB	0	100 ± 4		
	1st	100 ± 3	0	ns
	2nd	97 ± 2	- 3	ns
	3rd	94 ± 2	- 6	**
	5th	96 ± 3	- 4	ns
	10th	88 ± 3	- 12	***
	**Cleaning cycle for SDS/water**			
AcrA-MNPs+ SAB	0	100 ± 4		

	1st	100 ± 3	0	ns
	2nd	98 ± 2	- 2	ns
	3rd	98 ± 2	- 2	ns
	5th	98 ± 3	- 2	ns
	10th	98 ± 3	- 2	ns
	**Cleaning cycle for acetone/water**			
AcrA-MNPs+ SAB	0	100 ± 4		
	1st	96 ± 3	- 4	*
	2nd	92 ± 2	- 8	***
	3rd	89 ± 2	- 11	***
	5th	84 ± 4	- 16	***
	10th	76 ± 3	- 24	***

All data are expressed as mean ± standard deviation (n=3).

According to Student t-test *,**,*** and ns indicate significance at P < 0.001, 0.01, 0.05 and not significant, respectively.

Table S7. Protein removal efficiency of Milli-Q water, 10% SDS/water and acetone/water for Semillon wine treated with acrylic-acid plasma coated magnetic nanoparticles.

**Sample**	**Cleaning cycle for water**	**Removal efficiency (%)**	**Variation**	**Significance**
AcrA-MNPs+ SEM	0	100 ± 4		
	1st	95 ± 3	- 5	**
	2nd	93 ± 2	- 7	**
	3rd	90 ± 2	- 10	***
	5th	87 ± 3	- 13	***
	10th	85 ± 3	- 15	***
	**Cleaning cycle for SDS/water**			
AcrA-MNPs+ SEM	0	100 ± 4		
	1st	98 ± 3	- 2	ns
	2nd	97 ± 2	- 3	ns
	3rd	96 ± 2	- 4	**
	5th	96 ± 3	- 4	**
	10th	96 ± 3	- 4	**
	**Cleaning cycle for acetone/water**			
AcrA-MNPs+ SEM	0	100 ± 4		
	1st	85 ± 3	- 15	***
	2nd	85 ± 2	- 15	***
	3rd	83 ± 2	- 17	***
	5th	78 ± 4	- 22	***
	10th	75 ± 3	- 25	***

All data are expressed as mean ± standard deviation (n=3).

According to Student t-test *,**,*** and ns indicate significance at P < 0.001, 0.01, 0.05 and not significant, respectively.

Table S8. Protein removal efficiency of Milli-Q water, 10% SDS/water and acetone/water for Chardonnay wine treated with acrylic-acid plasma coated magnetic nanoparticles.

**Sample**	**Cleaning cycle for water**	**Removal efficiency (%)**	**Variation**	**Significance**
AcrA-MNPs+ CHA	0	100 ± 4		
	1st	100 ± 3	0	ns
	2nd	95 ± 2	- 5	**
	3rd	94 ± 2	- 6	**
	5th	93 ± 3	- 7	***
	10th	92 ± 3	- 8	***
	**Cleaning cycle for SDS/water**			
AcrA-MNPs+ CHA	0	100 ± 4		
	1st	100 ± 3	0	ns
	2nd	98 ± 2	- 2	ns
	3rd	98 ± 2	- 2	ns
	5th	98 ± 3	- 2	ns
	10th	98 ± 3	- 2	ns
	**Cleaning cycle for acetone/water**			
AcrA-MNPs+ CHA	0	100 ± 4		
	1st	94 ± 3	- 6	**
	2nd	90 ± 2	- 10	***
	3rd	88 ± 2	- 12	***
	5th	87 ± 4	- 13	***
	10th	86 ± 3	- 14	***

All data are expressed as mean ± standard deviation (n=3).

According to Student t-test *,**,*** and ns indicate significance at P < 0.001, 0.01, 0.05 and not significant, respectively.

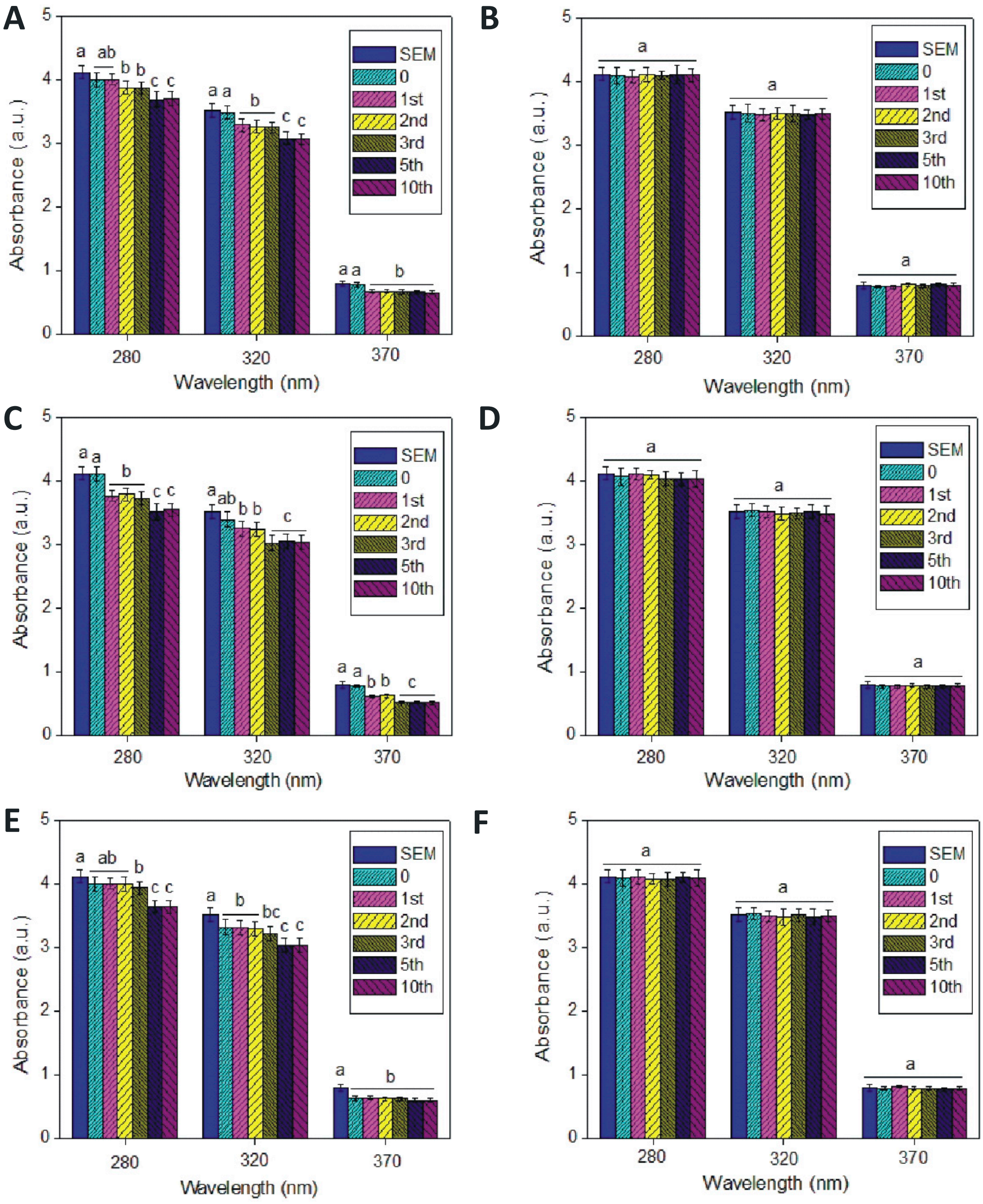

Figure 1. Phenolic content of Semillon wine before and after treatment with bare (A, C, E) and acrylic acid plasma coated magnetic nanoparticles (B, D, F). Three cleaning solvents were used: A and B Milli-Q water, C and D SDS, E and F acetone.

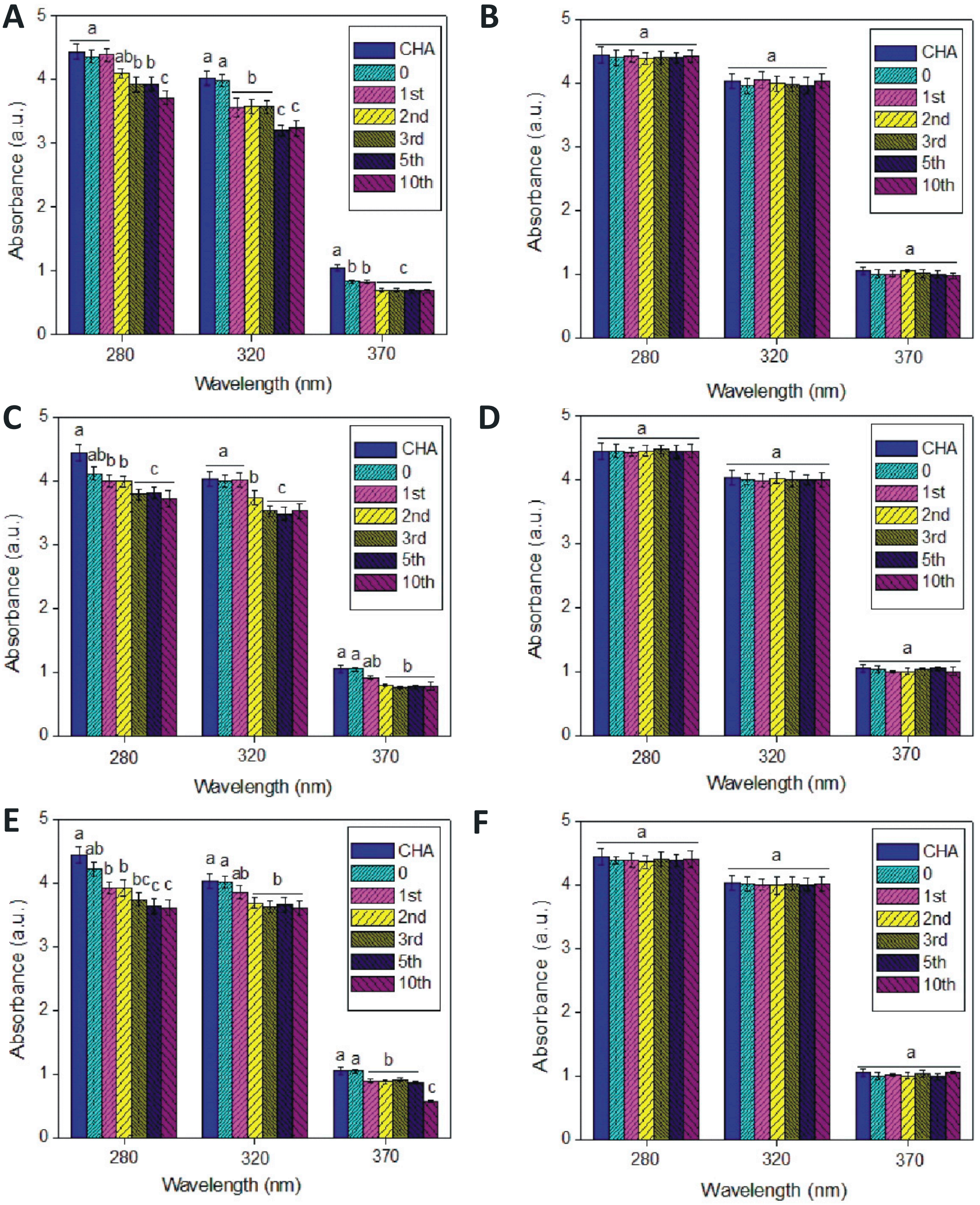

Figure S2. Phenolic content of Chardonnay wine before and after treatment with bare (A, C, E) and acrylic acid plasma coated magnetic nanoparticles (B, D, F). Three cleaning solvents were used: A and B Milli-Q water, C and D SDS, E and F acetone.

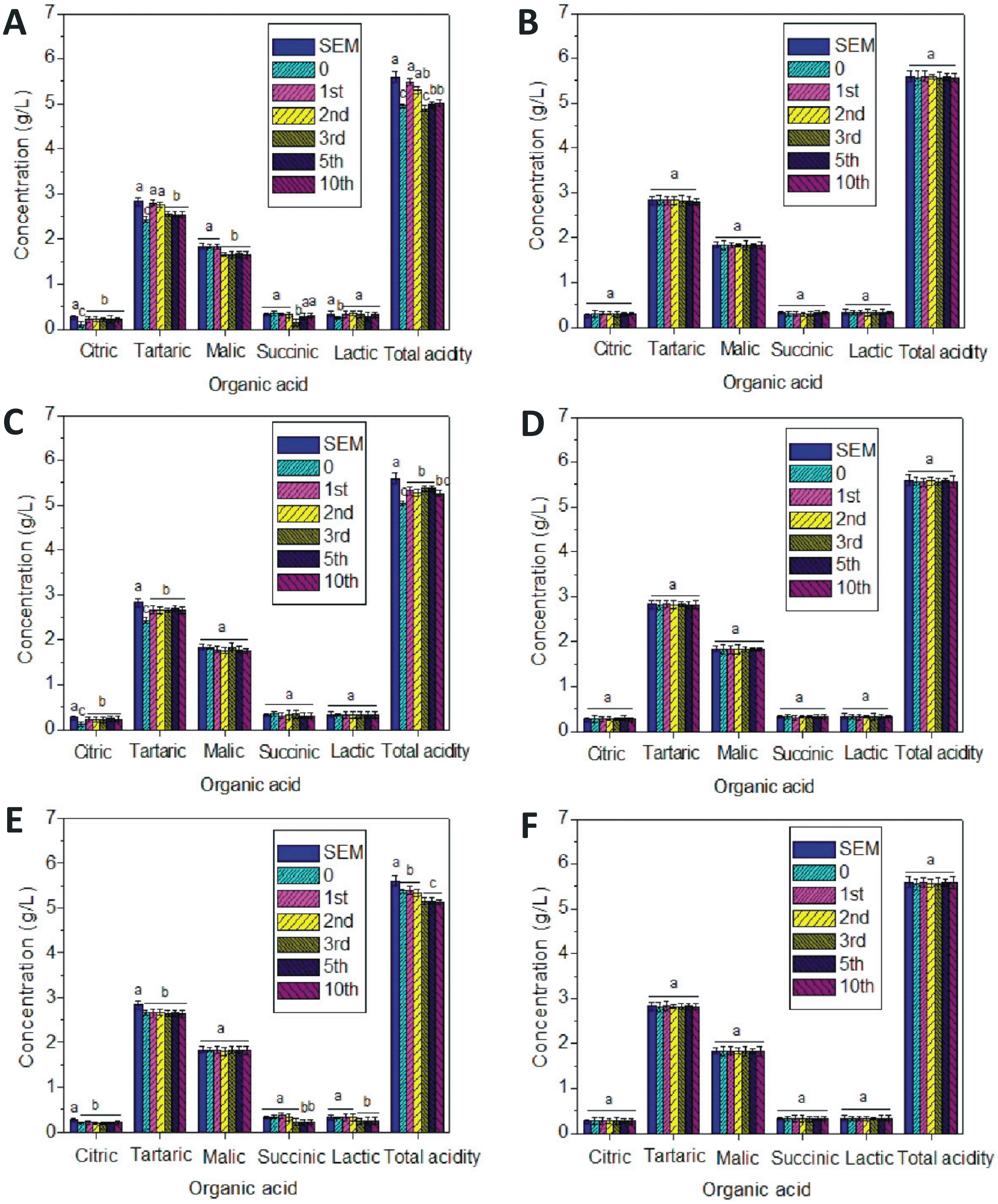

Figure S3. Organic acids concentration of Semillon wine before and after treatment with bare (A, C, E) and acrylic acid plasma coated magnetic nanoparticles (B, D, F). Three cleaning solution were used: A and B Milli-Q water, C and D SDS, E and F acetone.

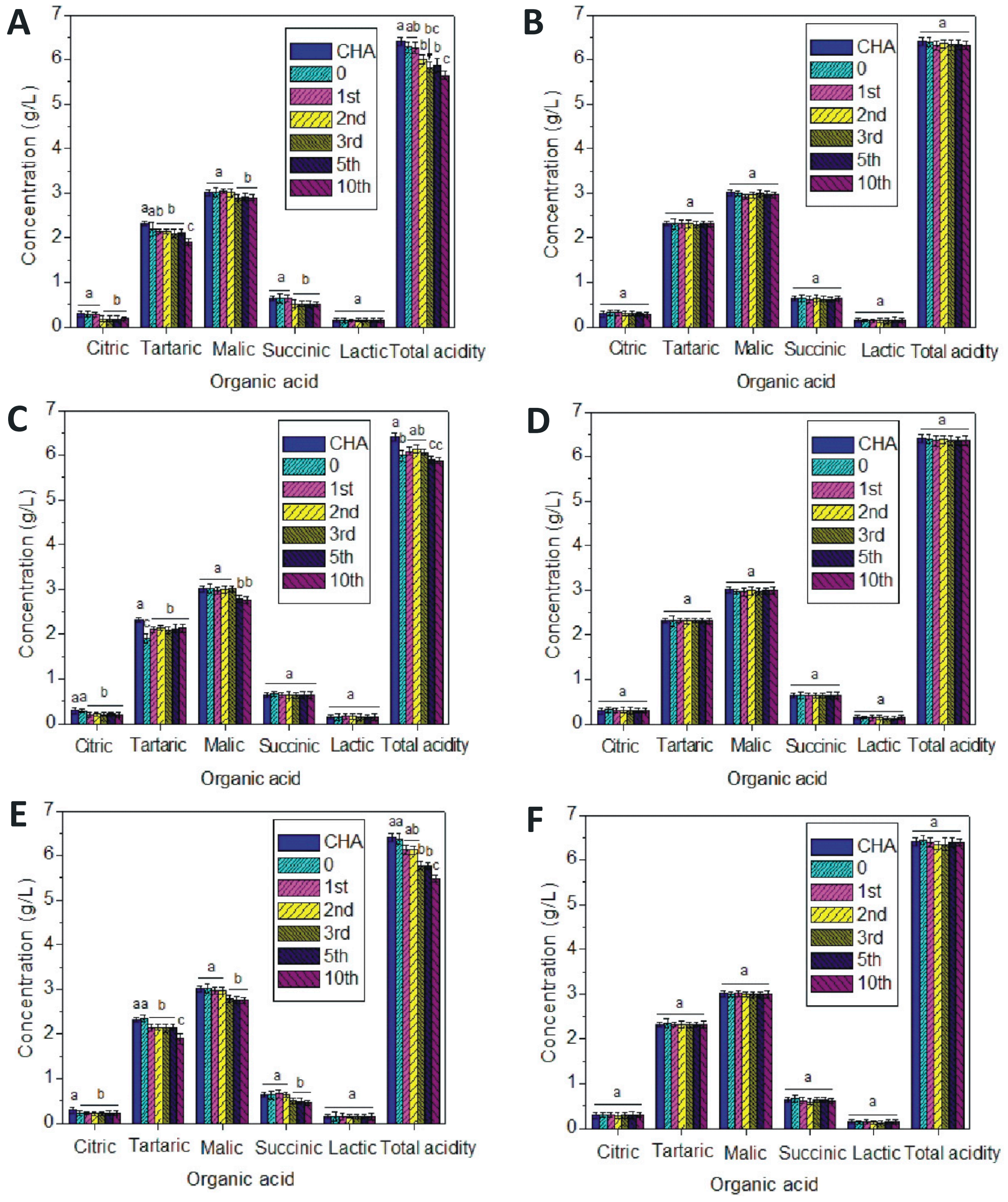

Figure S4. Organic acids concentration of Chardonnay wine before and after treatment with bare (A, C, E) and acrylic acid plasma coated magnetic nanoparticles (B, D, F). Three cleaning solution were used: A and B Milli-Q water, C and D SDS, E and F acetone.
